# Hsa-let-7d-5p Promotes Gastric Cancer Progression by Targeting PRDM5

**DOI:** 10.1155/2022/2700651

**Published:** 2022-07-07

**Authors:** Xiang Gao, Huiqi Liu, Rong Wang, Mingyu Huang, Qiong Wu, Yang Wang, Wei Zhang, Yongnian Liu

**Affiliations:** ^1^Research Centre for High Altitude Medicine, Qinghai University, Xining, Qinghai 810001, China; ^2^The Key Laboratory of Plateau Medicine Ministry of Education, Xining, Qinghai 810001, China; ^3^The Key Laboratory of High-Altitude Medical Application of Qinghai Province, Medical College of Qinghai University, Xining, Qinghai 810001, China; ^4^Research Center for Qinghai Healthy Development, Medical College, Qinghai University, Xining, Qinghai 810001, China; ^5^Department of Basic Medical Sciences, Medical College, Qinghai University, Xining, Qinghai 810016, China

## Abstract

Gastric cancer (GC) is a common malignant tumor in the digestive system and a significant health burden worldwide. In this study, we found that hsa-let-7d-5p was upregulated in GC cells, promoted GC cell proliferation, migration, and invasion, and reduced apoptosis. Moreover, we found that the expression of PRDM5 (PR domain protein 5) was downregulated in GC cells and upregulated in GC cells treated with hsa-let-7d-5p inhibitor. Further investigation showed that hsa-let-7d-5p was the target of PRDM5, and the functions of hsa-let-7d-5p on GC progression were rescued by PRDM5 overexpression in GC cells. Collectively, our findings suggested that hsa-let-7d-5p promoted the development of GC by targeting PRDM5, indicating that hsa-let-7d-5p could be a promising therapeutic molecule for the treatment of gastric cancer.

## 1. Introduction

Gastric cancer (GC) is a highly heterogeneous disease [1]. GC is characterized by rapid progression, poor prognosis, and low survival rate. Currently, the treatment of GC is unsatisfactory because the molecular mechanism of GC progression is not clear [2, 3]. Hence, it is urgent to investigate the regulating molecules in GC progression, which is beneficial to discover novel molecular targets for GC treatment [4–6].

MicroRNA (miRNA) was discovered in 1993 and subsequently researched in RNA biology [7]. MiRNAs are small noncoding RNAs that exert major roles in the posttranscriptional regulation of gene expression [8]. It was reported that miRNAs were the potent regulators of many cellular activities, including cell growth, proliferation, and apoptosis [7]. Moreover, they are related to the progression of multiple diseases, including neurodegenerative disorders [9], breast cancer [10], and GC [3]. Let-7 miRNA, discovered in *Caenorhabditis elegans*, is highly conserved in human tissues [11]. Let-7d-5p is one of the members of the 12 human let-7 family. It was known that the expression of let-7 was related to the development of aggressive cancers [12–14]. According to reports, the expression of let-7d is dysregulated in many types of cancer, such as HNSCC, breast cancer, renal cancer, retinoblastoma, pancreatic cancer, and prostate cancer. Let-7d plays a crucial role in cancer initiation, progression, and metastasis, where it functions as a tumor suppressor miRNA by regulating the expression of multiple oncogenes [15]. The miRNA expression of hsa-let-7d-5p was remarkedly increased in the exosomes of ovarian cancer patients compared to controls [16]. Let-7d-5p regulates the p53 signaling pathway through HMGA1 to inhibit ovarian cancer cell apoptosis [17]. Let-7d-5p can adjust HMGA1/Wnt/*β*-Catenin signaling to promote the growth and metastasis of colorectal cancer [18]. However, the molecular mechanism of let-7d-5p in GC remains unclear.

It was reported that PRDM (PR (PRDI-BF1 and RIZ) domain proteins) modulated cellular processes, such as differentiation, growth, and apoptosis [19], and dysfunction of PRDM was associated with genetic and epigenetic modifications in cancers [20]. PRDM5 (PR domain protein 5), a member of the PRDM family, acted as a new transcription repressor and a tumor suppressor in various human cancers [21, 22]. However, the regulatory mechanism of PRDM5 in GC needs to be investigated.

In this study, hsa-let-7d-5p was found to be significantly increased in human GC cell lines. In addition, knockdown of hsa-let-7d-5p promoted GC cell apoptosis and inhibited GC cell viability, migration, and invasion. The potential mechanism is that hsa-let-7d-5p promotes GC progression by targeting PRDM5. Our study will provide an experimental basis for the potential molecular mechanism of GC development and offer a novel therapeutic target against GC.

## 2. Materials and Methods

### 2.1. Cell Culture

The human GC cell lines (SGC-7901 and AGS) were obtained from the Chinese Academy of Sciences (Shanghai, China). AGS cells were cultured in F12K (51445C, SIGMA, St. Louis, MO, USA, N3520) with 10% FBS (10099, Thermo Fisher Scientific, USA) and NaHCO_3_ 2.5 g/L (Sigma, USA). SGC-7901 cells were cultured with RPMI-1640 (Bio-Channel, Nanjing, China) with 10% fetal calf serum (12484028, Thermo Fisher Scientific, Canada). The GES-1 normal gastric mucosal cell line (Beijing Institute of Cancer Research) was cultured in RPMI-1640 with 10% FBS. All the cells were maintained in an incubator at 37°C with 5% CO_2_.

### 2.2. Cell Transfection

Hsa-let-7d-5p mimic or the hsa-let-7d-5p inhibitor and its NC mimic or NC inhibitor, as well as PRDM5 siRNA (si-PRDM5) and its control siRNA (si-NC), were obtained from Genepharma, (Shanghai, China). For transfection, AGS and SGC-7901 cells were seeded into six-well plates (5 × 10^4^/well) and cultured overnight. Then, the cells were transfected with 200 nM hsa-let-7d-5p mimic, hsa-let-7d-5p inhibitor, or si-PRDM5 as well as their corresponding negative controls using Escort™ IV (Merck, Darmstadt, Germany).

### 2.3. CCK-8 Assay

Cell proliferation of AGS and SGC-7901 cells was measured using a CCK-8 kit (96992–500TESTS-F, Sigma-Aldrich, USA). At 0 h, 24 h, 48 h, and 72 h, each well was added CCK-8 solution and incubated for another 2 h at room temperature. Finally, cell viability was measured by an ultraviolet spectrophotometer (450 nm) (Thermo Fisher Scientific, Inc.).

### 2.4. Colony Formation Assay

AGS and SGC-7901 cells were seeded into 6-well plates (1 × 10^3^/well). After 2 weeks, all wells are washed with PBS three times, and the cell colonies were stained with 0.1% crystal violet at 37°C for 5 min. Then, the colonies were imaged and counted.

### 2.5. Cell Apoptosis Analysis

All cells were seeded in a 6-well plate (2 × 10^5^/well). After 24 h treatment, the cell apoptosis was analyzed by Annexin V-FITC apoptosis detection kit (Sigma-Aldrich, USA). AGS and SGC-7901 cells were harvested by centrifuging at 1000 rpm/min for 5 min. Then, cells (1 × 10^5^) were resuspended with 100 *μ*l buffer containing 5 *μ*l Annexin V-FITC and 5 *μ*l propidium (PI) at 4°C for 15 min in darkness. At last, cells were measured by flow cytometry (BD, Franklin Lake, NJ, USA). All the data were analyzed by FlowJo.

### 2.6. qRT-PCR Assay

Total RNA was isolated from AGS and SGC-7901 cells by TRIzol (Takara, Dalian, China). The reactions were carried out with SYBR® Green Master Mix (Vazyme, Nanjing, China). The thermal cycles were 95°C for 5 min, 35 cycles of 94°C for 35 s, and 62°C for 30 s. The primers for RT-qPCR were as follows: hsa-let-7d-5p: 5′-AGAGGUAGUAGGUUGCAUAGUU-3′ (forward) and 5′-CUAUGCAACCUACUACCUCUUU-3′ (reverse); PRDM5: 5′- CAGGTTCTCCCTGAAGTCCT-3′ (forward) and 5′-TGAGATGGTGCCTCATGAAC-3′ (reverse); hsa-U6: 5′-CTCGCTTCGGCAGCACA-3′ (forward) and 5′- AACGCTTCACGAATTTGCGT-3′ (reverse); GAPDH: 5′- AATGGGCAGCCGTTAGGAAA-3′ (forward) and 5′-TGAAGGGGTCATTGATGGCA-3′ (reverse). Hsa-U6 or GAPDH were used as the internal standards. The relative expression levels were assessed using the standard 2^−△△Ct^ method.

### 2.7. Western Blotting

Total protein from AGS and SGC-7901 cells was extracted by RIPA buffer (R0278-500 ML, Sigma-Aldrich). Samples were separated by 12% SDS-PAGE and transferred onto a PVDF membrane (Millipore, Billerica, USA) with 330 mA for 3.5 h. Then, TBST with 5% skim milk was used to block PVDF membranes, after that the PVDF membranes were incubated with primary antibodies against Bax (ab182733, 1 : 1500), Bcl-2 (ab59348, 1 : 2000), cleaved caspase-3 (ab214430, 1 : 1500), cleaved caspase-9 (ab2324, 1 : 1500), GAPDH (ab9485, 1 : 10000), and PRDM5 antibody (ab79016, 1 : 2000) (Abcam, Cambridge, UK) at 4°C overnight. Secondary antibodies (1 : 200; Abcam, Cambridge, UK) were incubated with PVDF membranes for 1 h at room temperature. PVDF membranes were visualized using an ECL detection reagent. The internal control was GAPDH.

### 2.8. Luciferase Reporter Assay

We implemented luciferase experiments in AGS and SGC-7901 cells to confirm whether PRDM5 was a target of has-let-7d-5p. WT or mutated (MUT) PRDM5 3′-UTR sequences were chemically synthesized (Sangon, Shanghai, China) according to the sequence of hsa-let-7d-5p. PRDM5-WT or PRDM5-Mut was, respectively, cotransfected with has-let-7d-5p mimic or inhibitor into AGS and SGC-7901 cells. Finally, luciferase activity was detected using the luciferase reporter assay system.

### 2.9. Transwell Invasion Assay

AGS and SGC-7901 cells were plated onto 24-well plates (1 × 10^4^/well) with the upper chamber with 100 *μ*l of Matrigel. The F12K medium or RPMI-1640 medium supplemented with 1% FBS was placed into the upper chamber. The F12K medium or RPMI-1640 medium with 10% FBS was placed into the lower chamber. After 24 h of incubation at 37°C, cells that invaded through the membrane were fixed with 5% glutaraldehyde at 4°C and stained with 0.1% crystal violet for 30 min. The cells were observed under a microscope (Dianying, Shanghai, China).

### 2.10. Cell Scratch Test

AGS and SGC-7901 cells were incubated in 6-well plates until 100% confluence. A sterile 10 *μ*L pipette tip was used to make a single scratch. The culture medium was replaced by an FBS-free F12K medium or RPMI-1640. Images of the scratches were captured at 0 and 48 h. Olympus CellSens Dimension software was used to measure the width of the scratch.

### 2.11. Statistical Analysis

All data were presented as the mean ± standard deviation (SD) and analyzed by Prism6. Comparisons were determined using unpaired/paired Student's *t*-test and one-way analysis of variance (ANOVA). The differences were deemed statistically significant at *P* < 0.05 (^*∗*^), *P* < 0.01 (^*∗∗*^), or *P* < 0.001 (^*∗∗∗*^).

## 3. Results

### 3.1. Let-7d-5p Is Upregulated in GC Cells, and Knockdown of Let-7d-5p Inhibits Cell Proliferation and Promotes Cell Apoptosis

**Figure 1:**
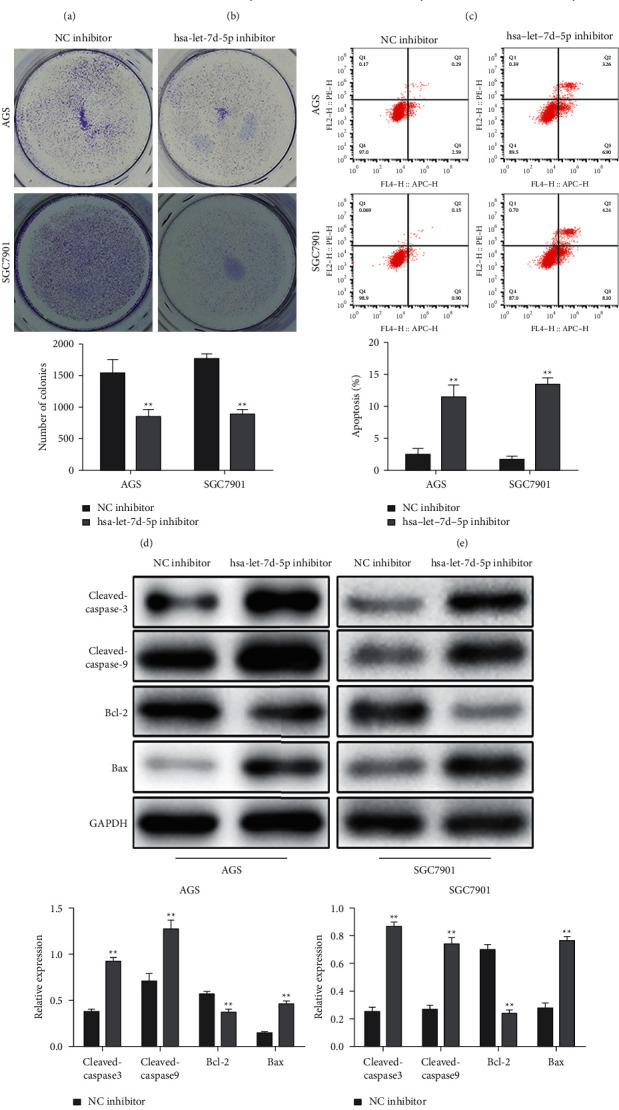
Let-7d-5p is overexpressed in GC cells, and knockdown of let-7d-5p inhibits proliferation of GC cells. AGS and SGC-7901 cells were transfected with a let-7d-5p inhibitor. (a) qRT-PCR tested the expression of let-7d-5p in GC cell lines (AGS and SGC-7901) and GSE-1 cells. Values are mean ± SD ^*∗∗*^*P* < 0.01 vs. GSE-1. *n* = 3 per group. (b) qRT-PCR analysis to detect the knockdown efficiency of let-7d-5p. (c) CCK-8 assays on the cell proliferation at the indicated time points in AGS and SGC-7901 cells (24, 48, and 72 h). (d) Colony formation assays in AGS and SGC-7901 cells. (e) Flow cytometry analyzed the apoptosis of cells. (f) The expression levels of antiapoptotic protein (Bcl-2) and proapoptosis proteins (Bax, cleaved caspase-3, and cleaved caspase-9) were detected by Western blot. Values are mean ± SD, ^*∗*^*P* < 0.05, ^*∗∗*^*P* < 0.01 vs. NC inhibitor, *n* = 3 per group.

To explore the biological function of let-7d-5p in human GC cells, we detected its expression in related cell lines. We found the upregulated expression of let-7d-5p in AGS and SGC-7901 cells compared with GES-1 by qRT-PCR analysis (Figure 1(a)). The knockdown efficiency of let-7d-5p in AGS and SGC-7901 cells was validated by qRT-PCR analysis. The results showed that let-7d-5p was downregulated by let-7d-5p inhibitor in GC cells (Figure 1(b)). Functionally, CCK-8 and colony formation assays showed that knockdown of let-7d-5p significantly inhibited the cell viability of GC cells (Figures 1(c) and 1(d)). Next, the flow cytometry assay revealed that knockdown of let-7d-5p promoted the apoptosis of AGS and SGC-7901 cells (Figure 1(e)). At the molecular level, the knockdown of let-7d-5p resulted in the downregulated expression of the antiapoptotic protein (Bcl-2). It elevated the expression levels of proapoptosis proteins (Bax, cleaved caspase-3, and cleaved caspase-9) in AGS and SGC-7901 cells (Figure 1(f)). These results indicated that knockdown of let-7d-5p inhibited cell proliferation and promoted the apoptosis of GC cells.

### 3.2. Low Expression of Let-7d-5p Inhibits the Migration and Invasion of GC Cells

**Figure 2:**
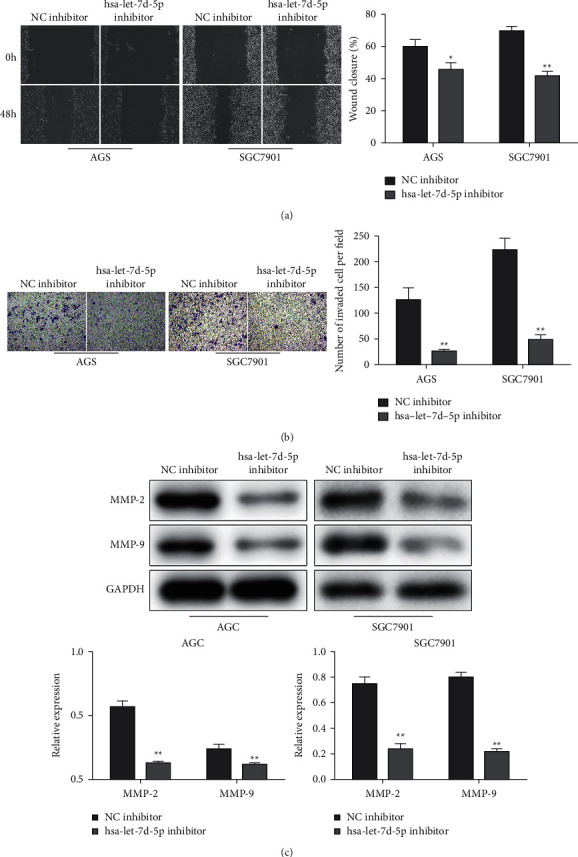
Knockdown of let-7d-5p inhibits migration and invasion of GC cells. AGS and SGC-7901 cells were transfected with a let-7d-5p inhibitor. ((a), (b)) Scratch test and transwell chamber assay for the migration and invasion abilities of AGS and SGC-7901 cells. (c) The MMP-2 and MMP-9 expression levels were analyzed by Western blot analysis. Values are mean ± SD, ^*∗*^*P* < 0.05, ^*∗∗*^*P* < 0.01 vs. NC inhibitor, *n* = 3 per group.

Transwell and scratch assays displayed that the migration and invasion abilities of AGS and SGC-7901 cells were remarkably restrained in the cells transfected with let-7d-5p inhibitor (Figures 2(a) and 2(b)). Also, the protein expression levels of matrix metalloproteinase-2 (MMP-2) and MMP-9, which are migration and invasion-related proteins, were downregulated by let-7d-5p inhibitor in AGS and SGC-7901 cells (Figure 2(c)). These results illustrated that the let-7d-5p inhibitor suppressed migration and invasion of GC cells.

### 3.3. PRDM5 Is a Target of Let-7d-5p

**Figure 3:**
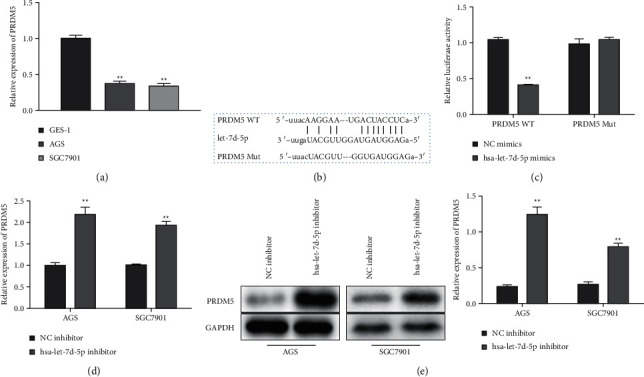
PRDM5 is the target of let-7d-5p, and let-7d-5p negatively regulates the expression of PRDM5. (a) The expression PRDM5 in AGS and SGC-7901 cells was analyzed by qRT-PCR. Values are mean ± SD ^*∗∗*^*P* < 0.01 vs. GSE-1. *n* = 3 per group. (b) Bioinformatic analysis of the predicted binding site between PRDM5 and let-7d-5p. (c) AGS and SGC-7901 cells were transfected with let-7d-5p mimic. Luciferase reporter assay detected the luciferase activity of PRDM5-WT in AGS and SGC-7901 cells. Values are mean ± SD, ^*∗∗*^*P* < 0.01 vs. NC mimic, *n* = 3 per group. ((d)-(e)) AGS and SGC-7901 cells were transfected with a let-7d-5p inhibitor. The expression of PRDM5 was analyzed by qRT-PCR and Western blot in GC cell lines (AGS and SGC-7901) and GSE-1 cells. Values are mean ± SD, ^*∗∗*^*P* < 0.01 vs. NC inhibitor, *n* = 3 per group.

Then, we explored the molecular mechanism of let-7d-5p in GC. Firstly, we found that the levels of PRDM5 were downregulated in AGS and SGC-7901 cells by qRT-PCR assay (Figure 3(a)). Thus, we used StarBase analysis to predict the potential interaction site between PRDM5 and let-7d-5p and mutated all binding sites (Figure 3(b)). To evaluate the interactions between PRDM5 and let-7d-5p, a luciferase reporter assay was performed and it revealed a significant decrease in luciferase activity in cells cotransfected with PRDM5-WT and let-7d-5p mimic (Figure 3(c)). In addition, the levels of PRDM5 were increased in AGS and SGC-7901 cells transfected with let-7d-5p inhibitor by qRT-PCR and Western blot (Figures 3(d) and 3(e)). These results indicated that let-7d-5p functioned by targeting PRDM5 in AGS and SGC-7901 cells.

### 3.4. Let-7d-5p Promotes Cell Proliferation and Inhibits Apoptosis through Regulating PRDM5 in GC

**Figure 4:**
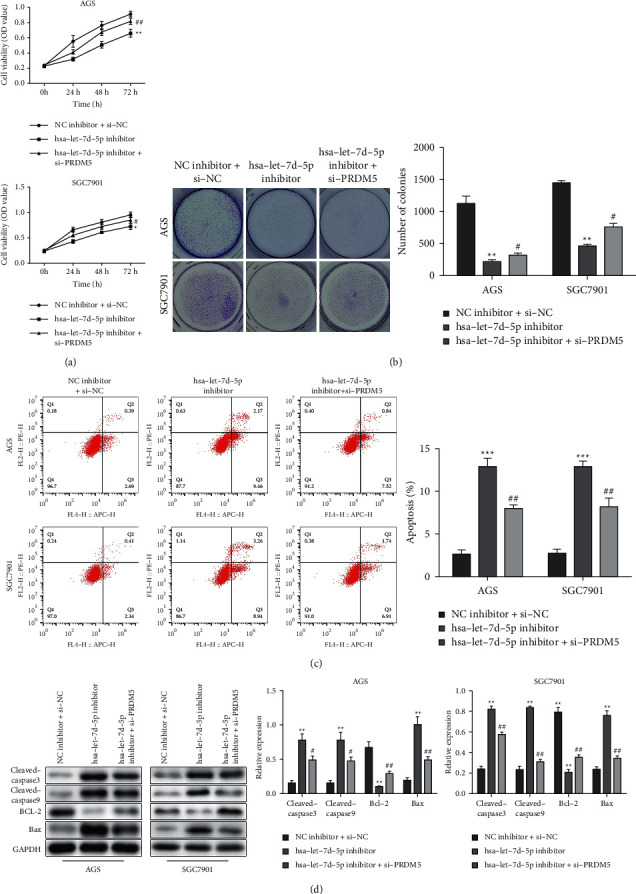
Let-7d-5p promotes GC cells proliferation and inhibits cell apoptosis through regulating PRDM5. Let-7d-5p inhibitor or/and si-PRDM5AGS were transfected into SGC-7901 cells. (a) CCK-8 assay to detect cell viability of AGS and SGC-7901 cells transfected with let-7d-5p inhibitor or/and si-PRDM5. (b) Colony formation assays for cell proliferation. (c) Flow cytometry analysis of the apoptosis rate. (d) The expression levels of antiapoptotic protein (Bcl-2) and proapoptosis proteins (Bax, Cleaved caspase-3, and Cleaved caspase-9) were detected by Western blot. Values are mean ± SD, ^#^*P* < 0.05, ^##^*P* < 0.01 vs. let-7d-5p inhibitor, ^*∗*^*P* < 0.05, ^*∗∗*^*P* < 0.01 vs. NC inhibitor + si-NC, *n* = 3 per group.

The CCK-8 assay and colony formation assay illustrated that si-PRDM5 reversed the inhibitory effect of the let-7d-5p inhibitor on cell viability and proliferation of AGS and SGC-7901 cells (Figures 4(a) and 4(b)). Then, apoptosis assay was performed by flow cytometry and showed that si-PRDM5 rescued the promotive effects of the let-7d-5p inhibitor on apoptosis in AGS and SGC-7901 cells (Figure 4(c)). Moreover, Western blot analysis revealed that si-PRDM5 rescued the downregulated antiapoptotic protein (Bcl-2) and suppressed the elevation of proapoptosis proteins (Bax, cleaved caspase-3, and cleaved caspase-9) caused by let-7d-5p inhibitor in AGS and SGC-7901 cells (Figure 4(d)). These results demonstrated that si-PRDM5 could reverse the function of let-7d-5p inhibitor on AGS and SGC-7901 cells, suggesting that let-7d-5p promotes GC cell proliferation and inhibits cell apoptosis through PRDM5.

### 3.5. Let-7d-5p Promotes Migration and Invasion of GC Cells through Regulating PRDM5

**Figure 5:**
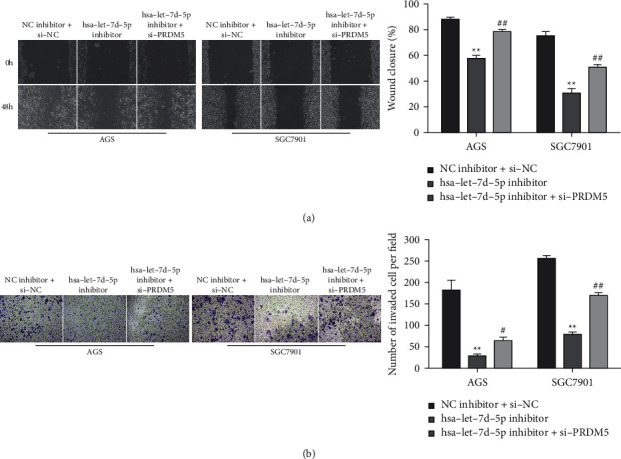
Let-7d-5p promotes migration and invasion of GC cells through regulating PRDM5. AGS and SGC-7901 cells were transfected with let-7d-5p inhibitor or/and si-PRDM5. ((a), (b)) Scratch test and transwell chamber assay for the migration and invasion abilities in AGS and SGC-7901 cells. Values are mean ± SD, ^##^*P* < 0.01 vs. let-7d-5p inhibitor, ^*∗∗*^*P* < 0.01 vs. NC inhibitor + si-NC, *n* = 3 per group.

Scratch and transwell chamber assays revealed that si-PRDM5 rescued repressive effects of the let-7d-5p inhibitor on the migration and invasion abilities of AGS and SGC-7901 cells (Figures 5(a) and 5(b)). These results demonstrated that si-PRDM5 could reverse the suppression of let-7d-5p inhibitor on AGS and SGC-7901 cells, suggesting that let-7d-5p promotes GC cell migration and invasion through PRDM5.

## 4. Discussion

GC represents the fifth cause of cancer-related deaths worldwide [23]. MiRNAs have been found to participate in the pathogenesis of GC and could be promising therapeutic targets in cancers [24, 25]. Therefore, it is worth exploring the underlying regulation of miRNAs in the development of diseases and using them as therapeutic targets in clinical applications.

Noncoding RNAs (ncRNAs) are associated with many human diseases [26]. MiRNAs regulate target gene expression by binding to the 3′ UTR region of the target gene and are involved in the pathogenic processes of various human diseases, including GC [27, 28]. The analysis of miRNA and gene expression profiles showed that the let-7 miRNA family plays a central role in the regulation of tumorigenesis [29, 30]. And the let-7 family members are the hallmarks of several cancers [31, 32]. However, the downstream regulatory molecules of let-7d-5p in GC progression are rarely reported. In this study, let-7d-5p was upregulated in GC cells. Low expression of let-7d-5p suppressed cell proliferation, migration, and invasion and promoted cell apoptosis of GC cells.

Recent studies have highlighted the association of PRDM5 with the progression of human cancers [19, 33, 34]. PRDM5 is a sequence-specific transcriptional repressor [35]. It has been shown that PRDM5 is silenced by promoter methylation in multiple cancers [36]. Few studies showed that PRDM5 was targeted by noncoding RNAs. It has been demonstrated that PRDM5 was targeted by miR-130b-5p, and BDNF-AS/miR-130b-5p/PRDM5 axis worked in the development of acute spinal cord injury [37]. In our study, we found that PRDM5 expression was negatively regulated by let-7d-5p in GC cells. Additionally, we found that the knockdown of PRDM5 reversed the suppression of let-7d-5p inhibitor in cell proliferation, migration, and invasion and the promotion of cell apoptosis in GC cells. These results suggested that there was an interaction between let-7d-5p and PRDM5 in GC cells. We performed rescue experiments to explore the regulation mechanism of cell apoptosis, proliferation, migration, and invasion between let-7d-5p and PRDM5. It was confirmed that si-PRDM5 partially antagonized the let-7d-5p inhibitor-mediated effects on cell apoptosis, proliferation, migration, and invasion in GC cells.

## 5. Conclusion

In summary, this study demonstrated that knockdown of let-7d-5p could promote cell apoptosis and inhibited cell invasion and migration in GC cells. There was a regulatory interaction between let-7d-5p and PRDM5 in GC cells. The complex of let-7d-5p and PRDM5 could become a promising therapeutic target for the treatment of GC.

## Data Availability

All data generated or analyzed during this study are included in this published article.

## Abbreviations

GC: Gastric cancer
PRDM5: PR domain protein 5
PRDM: PR (PRDI-BF1 and RIZ) domain proteins
PVDF: Polyvinylidene fluoride
MMP-2: Matrix metalloproteinase-2
MMP-9: Matrix metalloproteinase-9
Bcl-2: B-cell lymphoma-2
Bax: Bcl-2-associated X.
